# Influence of pregnancy/childbirth on long-term bone marrow edema and subchondral sclerosis of sacroiliac joints

**DOI:** 10.1007/s00256-020-03700-9

**Published:** 2021-01-21

**Authors:** Christoph Germann, Daniela Kroismayr, Florian Brunner, Christian W. A. Pfirrmann, Reto Sutter, Veronika Zubler

**Affiliations:** 1 Radiology, Balgrist University Hospital, University of Zurich, Forchstrasse 340, CH-8008 Zurich, Switzerland; 2 Physical Medicine and Rheumatology, Balgrist University Hospital, University of Zurich, Forchstrasse 340, CH-8008 Zurich, Switzerland

**Keywords:** Magnetic resonance imaging, Postpartum period, Sacroiliac joint, Sacroiliitis, Spondyloarthritis

## Abstract

**Objective:**

To investigate long-term effects of pregnancy/childbirth on bone marrow edema (BME) and subchondral sclerosis of sacroiliac joints (SIJ) in comparison to MRI changes caused by spondyloarthritis (SpA) and assess the influence of birth method and number of children on SIJ-MRI changes.

**Materials and methods:**

This is a retrospective cohort study with 349 women (mean age 47 ± 14 years) suffering low back pain. Four subgroups were formed based on SpA diagnosis and childbirth (CB) history. Two musculoskeletal radiologists scored the presence of BME and sclerosis on SIJ-MRI using the Berlin method. Further, an 11-point “global assessment score” representing the overall confidence of SpA diagnosis based on MRI was evaluated in addition to the ASAS (Assessment of Spondyloarthritis International Society) criterion of “positive MRI” for sacroiliitis.

**Results:**

CB did not correlate with BME score (*p* = 0.38), whereas SpA diagnosis was associated with a higher BME score (*r* = 0.31, *p* < 0.001). Both CB (*r* = 0.21, *p* < 0.001) and SpA diagnosis (*r* = 0.33, *p* < 0.001) were correlated with a higher sclerosis score. CB was not associated with a higher confidence level in diagnosing SpA based on MRI (*p* = 0.07), whereas SpA diagnosis was associated with a higher score (*r* = 0.61, *p* < 0.001). Both CB (phi = 0.13, *p* = 0.02) and SpA diagnosis (phi = 0.23, *p* < 0.001) were significantly associated with a positive ASAS criterion for sacroiliitis. In non-SpA patients with CB, number of children (*p* = 0.001) was an independent predictor of sclerosis score, while birth method yielded no significant effect (*p* = 0.75).

**Conclusion:**

Pregnancy/CB has no impact on long-term BME on SIJ, however, may cause long-term subchondral sclerosis—similar to SpA-associated sclerosis. Number of children is positively correlated with SIJ sclerosis. Birth method yields no effect on SIJ sclerosis.

## Introduction

Low back pain is a common health problem in the general population with a point prevalence of 12-33% [1, 2]. The differential diagnosis is broad, particularly comprised degenerative, mechanical (e.g., stress-related), traumatic, and inflammatory pathologies. Low back pain originates in particular from either the lumbar spine or the sacroiliac joints (SIJ). It may be difficult to distinguish various causes of low back pain based merely on history and/or clinical examination and further diagnostic work-up is often needed. Hence, due to the high prevalence of low back pain, a substantial number of patients will undergo MR imaging. Especially in the young to middle-aged adult population, important differential diagnoses are inflammatory pathologies such as axial spondyloarthritis (axSpA), manifesting often as sacroiliitis [3]. Typical MRI changes in axSpA are active inflammation, expressed by bone marrow edema (BME), and structural changes such as subchondral sclerosis, fatty bone marrow replacement, erosions, and ankylosis of the SIJ [4, 5]. It is known that non-inflammatory causes of back pain, e.g. due to lumbar disc herniation [6–8], as well as mechanical stress on the pelvic girdle, e.g. caused by pregnancy/childbirth or physical activity in athletes can cause subchondral BME and subchondral sclerosis of the SIJ which may be indistinguishable from axSpA [9–15]. This often poses a diagnostic dilemma in female patients with a history of pregnancy and childbirth, because overcalling these findings would lead to unnecessary treatment and costs, whereas to consider them as always normal after childbirth may delay treatment and reduce patient outcome in cases of actual axSpA.

Very recently it has been shown that pregnancy/delivery can cause persisting SIJ-subchondral sclerosis in the late postpartum period (≥ 2 years after childbirth), whereas pregnancy-/birth-related BME along the SIJ seems to vanish after the first postpartum year [16]. However, not yet addressed issues are as follows: (a) if SpA-related subchondral sclerosis differs from pregnancy/birth-related subchondral sclerosis in the late postpartum period and (b) if birth method (vaginal delivery or cesarean surgery) and/or number of children have an impact on the extent of sclerosis.

Therefore, the purpose of this study was to investigate the long-term (more than 24 months after last childbirth) effect of pregnancy/childbirth on BME and subchondral sclerosis of SIJ in comparison to SpA-induced changes; and furthermore, to test if the birth method (vaginal delivery or cesarean surgery) and/or number of children have an impact on these SIJ-MRI changes.

## Materials and methods

### Study population

This retrospective single-center cohort study was approved by the local ethics committee. All included patients have given written informed consent and completed a questionnaire regarding their obstetric history at the time of MRI acquisition: number of children, birth method (vaginal delivery/C-section), and date of the last delivery.

**Fig. 1:**
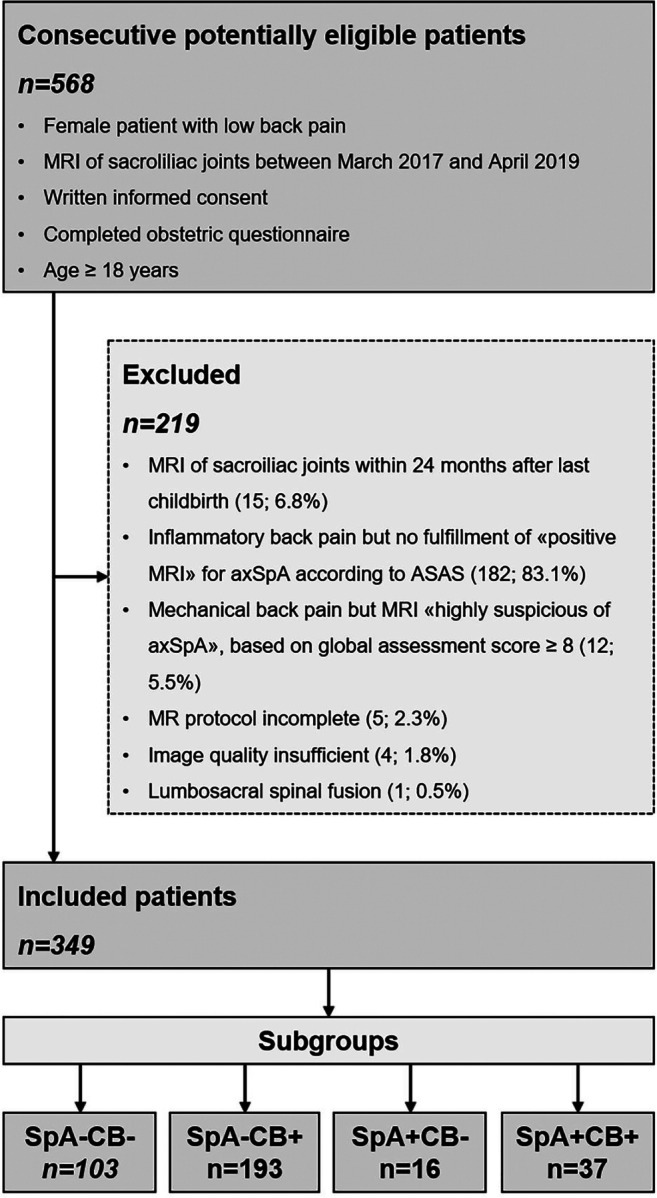
Flowchart of study design and patient inclusion/exclusion. Fulfillment of ASAS criteria of a “positive MRI” for axSpA: either one BME lesion on two or more consecutive slices or more than one BME lesion on a single slice [17]. Global assessment score: subjective ordinal scale (range 0 to 10) based on the diagnostic certainty of the radiologist for axSpA: score 0 means the radiologist has no MRI evidence of axSpA at all, whereas a score of 10 signifies absolute certainty that the respective patient does have axSpA; we defined a score of ≥ 8 as “highly suspicious of axSpA”. Both these criteria were applied to prevent false-negative and false-positive misclassification. *ASAS* Assessment of Spondyloarthritis International Society, ax*SpA* axial spondyloarthritis, *CB* childbirth, *SIJ* sacroiliac joint. Subgroups: *SpA*−*CB*− no SpA and no CB history, *SpA*−*CB+* no SpA with CB history, *SpA+CB*− SpA without CB history, *SpA+CB+* patients with SpA and CB history

Inclusion criteria were as follows: (1) female patient with low back pain referred to our radiology department for MR imaging of the SIJ from March 2017 to April 2019 by a board-certified orthopedic surgeon or rheumatologist (after clinical history was taken and physical examination was performed); (2) written informed consent; (3) completed obstetric questionnaire; and (4) age ≥ 18 years. Exclusion criteria comprised (1) SIJ-MRI within 24 months after last childbirth; (2) patients with inflammatory-type back pain but without fulfillment of the Assessment of Spondyloarthritis International Society (ASAS) criteria for classification of axSpA [17]; (3) patients with mechanical-type low back pain but MRI findings highly suspicious of axSpA—based on a global assessment score ≥ 8 (for definition of global assessment score, see the “Definition of MR imaging features” section within the “Materials and methods” section); (4) incomplete MRI dataset; (5) insufficient image quality; and (6) previous lumbosacral spinal fusion. Figure 1 shows a detailed flowchart of patient inclusion/exclusion.

SpA diagnosis was based on a combination of clinical examination (by a board-certified rheumatologist: e.g., inflammatory back pain, positive family history), blood tests (i.e., CRP and HLA-B27), and sacroiliitis on imaging (interpretation by a board-certified radiologist), fulfilling standardized ASAS criteria for classification of axial spondyloarthritis [4, 17].

Based on the two variables childbirth (CB) and axSpA, our cohort was categorized in 4 subgroups: (1) no SpA/no childbirth (SpA−CB−); (2) no SpA/childbirth (SpA−CB+); (3) SpA/no childbirth (SpA+CB−); and (4) SpA/childbirth (SpA+CB+).

### MRI

MRI examinations of the SIJ at our institution were performed on 1.5 T or 3 T systems (MAGNETOM Avanto fit or MAGNETOM Skyra fit, Siemens Healthcare, Erlangen, Germany) using a combination of an 18-channel surface and a 32-channel spine transmit/receive coil. The standardized protocol comprised a coronal oblique short tau inversion recovery (STIR), a coronal oblique T1-weighted sequence—both orientated strictly perpendicular to the upper endplate of S1—and an oblique axial STIR sequence. Detailed sequence parameters are shown in Table 1.

**Table 1:** Dedicated SIJ-MRI protocol at 1.5 T and 3 T

	Sequence	Plane	FOV (mm)	Matrix	Slice thickness (mm)	Interslice distance (mm)	TE/TR (ms)	TA (min:s)
1.5 T	T1	Obl cor	220 × 220	320 × 288	2	0.4	11/529	03:45
	STIR	Obl cor	240 × 240	320 × 256	4	0.4	57/4000	03:24
	STIR	Obl ax	240 × 240	320 × 256	4.5	0.7	48/4000	03:24
3 T	T1	Obl cor	220 × 220	384 × 269	1.5	0.3	13/727	05:08
	STIR	Obl cor	220 × 220	384 × 307	4	0.4	39/6680	03:47
	STIR	Obl ax	220 × 220	384 × 288	4	0.4	38/4180	03:58

*FOV* field of view, *SIJ* sacroiliac joints, *STIR* short tau inversion recovery, *TA* acquisition time, *TE* echo time, *TR* repetition time, *obl* oblique, *cor* coronal, *ax* axial

### Image analysis

All MR images were retrospectively evaluated by a fellowship-trained musculoskeletal radiologist (C.G.) with 2 years of experience in specialized musculoskeletal radiology. Additionally, one-third of all included image datasets were randomly selected and assessed by a second musculoskeletal-subspecialized radiologist (V.Z.) with 10 years of experience to test for inter-reader agreement. Evaluations were performed in an independent and randomized fashion on anonymized datasets following the removal of personal and clinical information using state-of-the-art picture archiving and communication system (PACS) workstations.

**Fig. 2:**
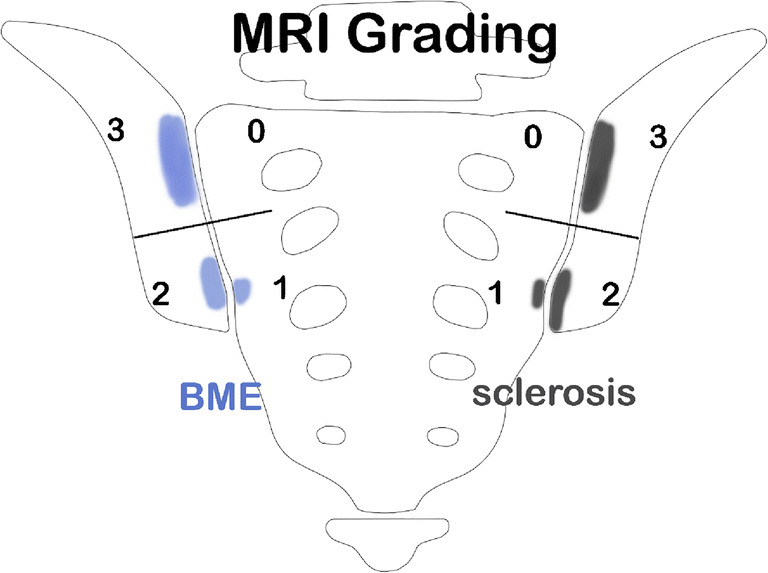
Oblique coronal view on SIJ with illustration of quadrant-based grading system (Berlin method). Both SIJ are divided into 4 quadrants, respectively, each of which is graded separately in a semiquantitative manner in regard to presence of BME and sclerosis: not present (score = 0), < 33% of quadrant area (score = 1), ≥ 33 to <66% of quadrant area (score = 2), and ≥ 66% of quadrant area (score = 3). Right SIJ depicts BME score 0 to 3, left SIJ depicts sclerosis score 0 to 3, respectively. *BME* bone marrow edema, *SIJ* sacroiliac joint

Both SIJ were evaluated regarding BME and subchondral sclerosis using the established semiquantitative “Berlin method,” which has been used in various studies and shown its capability to accurately assess SIJ-MRI findings similar to other more detailed scoring systems such as the Spondyloarthritis Research Consortium of Canada (SPRCC) MRI index or SPRCC MRI structural score [4, 9, 18, 19]. The “Berlin method” is based on global grading of the cartilaginous portion of the SIJ [19–21]: Each SIJ was divided into 4 quadrants (upper ilium, lower ilium, upper sacrum, lower sacrum), respectively, and each quadrant was graded separately as follows: BME/sclerosis not present: score = 0; < 33% of quadrant area: score = 1; ≥ 33 to <66% of quadrant area: score = 2; and ≥ 66% of quadrant area: score = 3 (Fig. 2). Consequently, the range for scoring was 0-24 points per patient (composed of a maximum of 12 points per side) for each parameter. Additionally, the maximum depth of subchondral sclerosis on the iliac and the sacral side was measured and expressed as the sum of the largest width perpendicular to the joint surface on both sacral and iliac sides.

### Definition of MR imaging features

In order to facilitate legibility of the manuscript, we decided to use the term “BME” rather than “edema-like marrow signal intensity” or “bone marrow edema-like signal intensity” which more precisely reflects the actual histopathologic findings [22].

Subchondral BME was defined as (1) focal hyperintense signal on STIR images and (2) location in the periarticular region, i.e., in contact with the subchondral bone plate [4, 23].

Subchondral sclerosis was defined as an area of low signal intensity in both T1-weighted and STIR sequences adjoining the cartilaginous portion of the SIJ [4].

According to the ASAS definition of a “positive MRI” for sacroiliitis, we classified each patient based on the presence of BME as either “positive” or “negative”: “ASAS positive” signifies either one BME lesion on two or more consecutive slices or more than one BME lesion on a single slice [5, 17, 24].

Accounting for the low specificity of an “ASAS positive MRI for sacroiliitis” regarding diagnosing SpA, we included a “global assessment score” in our evaluation, using a subjective ordinal scale (range 0 to 10) based on the diagnostic certainty of the radiologist: A score 0 means the radiologist found absolutely no MRI evidence of axSpA, whereas a score of 10 signifies absolute certainty that the respective patient does have axSpA. For this interpretation, all possible MRI features of axSpA were considered: BME, sclerosis, fat metaplasia, erosions, and ankylosis; however, no quantitative systematic analysis of fat metaplasia, erosions, or ankylosis was performed.

### Statistical analysis

Statistical analysis was performed using SPSS (v25, IBM Corp., Somers, NY). General descriptive statistics were applied. Categorical data are presented as proportions/percentages and continuous data as means with standard deviations. Inter-reader agreement between both radiologists was assessed using Cohen’s kappa for qualitative variables and intraclass correlation coefficient (ICC) for continuous variables. Kappa values were interpreted according to Kundel and Polansky [25], ICC values according to Koo and Li [26].

Kolmogorov-Smirnov test was applied to test for normal distribution. Age between groups was compared using analysis of variance (ANOVA) with Bonferroni correction. Kruskal-Wallis test was applied in subgroup analysis regarding non-normal distributed continuous variables (BME score, sclerosis score, sclerosis depth and subjective score). Localization of BME and sclerosis (upper or lower half of SIJ) within each subgroup was compared using Wilcoxon-signed-rank test.

We correlated age, time since last childbirth, number of children, and birth method (vaginal delivery; C-section; both) with BME score, sclerosis score, depth of sclerosis, global assessment score, and ASAS grade using Pearson’s correlation coefficient, eta coefficient, and phi coefficient, respectively. In case of statistically significant correlation, an additional multivariable regression analysis was performed. A *p* value < 0.05 was considered to represent statistical significance.

## Results

### Inter-observer reliability

Inter-observer agreement was “substantial” for ASAS score (*κ* = 0.63); “good” for sclerosis score (ICC = 0.82) and sclerosis depth (ICC = 0.83); and “excellent” for global assessment score (ICC = 0.93) and total BME score (ICC = 0.95).

### Demographics

Based on the recruitment criteria (Fig. 1), a total of 349 female patients with a mean age of 47 ± 14 years (range 18-87 years) were included. Two hundred thirty of 349 (66%) subjects have had at least one childbirth in their history as opposed to 119 of 349 (34%) without childbirth. Fifty-three of 349 (15%) included patients have SpA (SpA+), 296 of 349 (85%) do not have SpA (SpA−), 207 of 3494 (59%) patients had a 1.5 T MRI, and 142 of 349 (41%) had a 3 T MRI of the sacroiliac joints. Four subgroups were formed based on the criteria SpA and childbirth: SpA−CB− (*n* = 103), SpA−CB+ (*n* = 193), SpA+CB− (*n* = 16), and SpA+CB+ (*n* = 37), respectively. The magnetic field strength ratio (1.5 T/3 T MRI) was evenly spread in all subgroups: SpA−CB− 59%/41%, SpA−CB+ 59%/41%, SpA+CB− 62%/38%, and SpA+CB+ 59%/41%, *p* = 0.99.

The portion of patients with a BME score of ≥ 1 in the entire cohort was 51.3% (179/349), and the percentage with a sclerosis score of ≥ 1 was 59.9% (209/349). In the four subgroups, a BME score of ≥ 1 was seen in 42.7% for group SpA−CB−, in 51.8% for SpA−CB+, in 68.7% for SpA+CB−, and in 64.9% for SpA+CB+. Accordingly, a sclerosis score of ≥ 1 was given in 39.8% (group SpA−CB−), 64.2% (SpA−CB+), 75.0% (SpA+CB−), and 86.5% (SpA+CB+), respectively.

Patients in subgroup SpA−CB+ were 51 ± 12 years old and in regard to childbirth, 69.4% of patients (134/193) had exclusively vaginal deliveries, 18.7% (36/193) only C-sections, and 11.9% (23/193) at least one of each. The mean number of children in subgroup SpA−CB+ was 2.1 ± 1.0 (range 1-7), and the average time between MRI and last childbirth was 254.7 ± 155.4 months (range 27-753 months). In subgroup SpA+CB+, the mean age was 48 ± 12 years, and 59.5% of patients (22/37) had only vaginal deliveries, 24.3% (9/37) only C-sections, and 16.2% of patients (6/37) at least one of each. The number of children in subgroup SpA+CB+ was 2.4 ± 1.4 (range 1-7) and the average time between MRI and last childbirth was 220.6 ± 143.0 months (range 25-549 months).

### Correlation analysis in the entire cohort (*n* = 349)

Table 2 shows the influence of age, CB, and SpA diagnosis on BME, sclerosis, overall assessment score, and ASAS score.

**Table 2:**
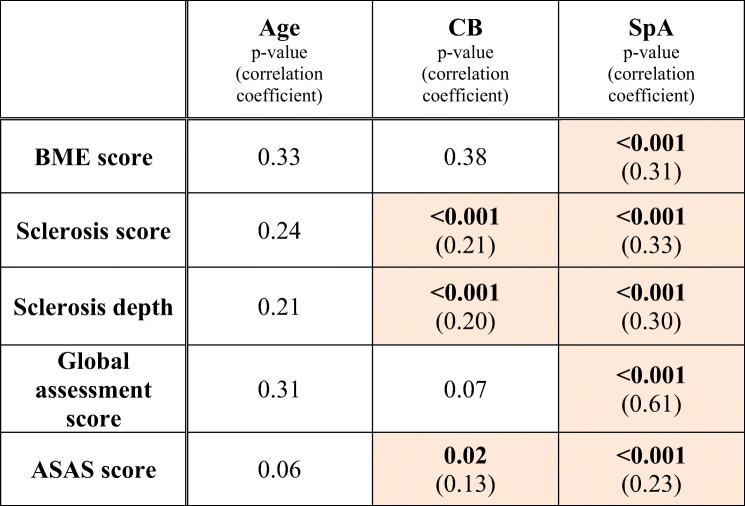
Correlation analysis in the entire cohort (*n* = 349)

Numbers represent *p* values. If *p* value is < 0.05, the correlation coefficient is given in parenthesis. The color-highlighted values are statistically significant correlations

*ASAS* Assessment of Spondyloarthritis International Society, *BME* bone marrow edema, *CB* childbirth, *SpA* spondyloarthritis

**Fig. 3:**
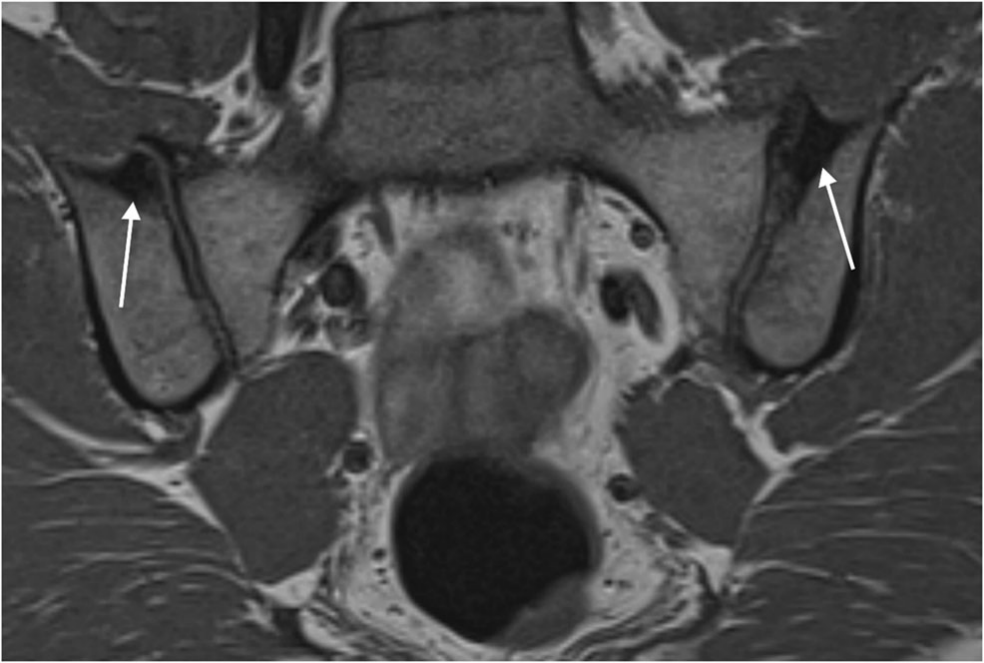
Oblique coronal T1-weighted MRI of SIJ of a 47-year-old woman with mechanical low back pain (no SpA) and childbirth history: two children, one of which delivered naturally, the other by C-section. The last delivery was 22 years and 8 months ago. Image depicts substantial subchondral sclerosis (arrows) in the upper quadrant of both SIJ in the os ilium, representing a sclerosis score of 2 on the right side and 3 on the left side, respectively. *C-section* cesarean surgery, *SIJ* sacroiliac joint, *SpA* spondyloarthritis

Age and CB did not correlate with BME score (*p* = 0.33; *p* = 0.38, respectively), whereas SpA diagnosis was positively associated with BME score (*r* = 0.31, *p* < 0.001). Age showed no correlation with sclerosis score (*p* = 0.24) or sclerosis depth (*p* = 0.21); however, both CB and SpA diagnosis were positively associated with sclerosis score (*r* = 0.21; *r* = 0.33; *p* < 0.001, respectively) and sclerosis depth (*r* = 0.20; *r* = 0.30; *p* < 0.001, respectively) (Fig. 3). Age (*r* = 0.05; *p* = 0.31) and CB (*r* = 0.1; *p* = 0.07) did not correlate with global assessment score, as opposed to a significant positive correlation between SpA diagnosis and global assessment score (*r* = 0.61; *p* < 0.001). Age showed no association with ASAS score (*r* = 0.1; *p* = 0.06), whereas both CB (phi = 0.13; *p* = 0.02) and SpA diagnosis (phi = 0.23; *p* < 0.001) were associated with a positive ASAS score.

### Subgroup comparison

**Fig. 4:**
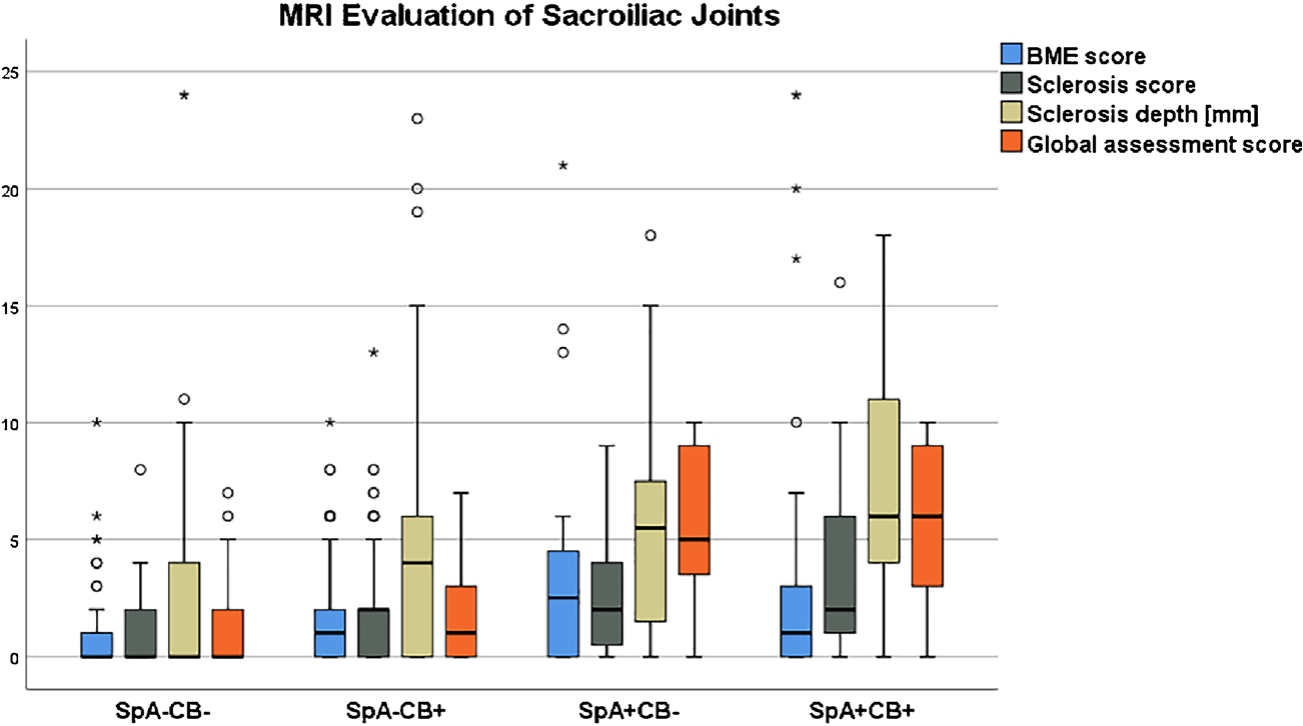
Boxplot of BME score (0-24), sclerosis score (0-24), sclerosis depth (in mm), and global assessment score (0-10) in the four different subgroups: *SpA*−*CB*− no SpA and no CB history, *SpA*−*CB+* no SpA with CB history, *SpA+CB*− SpA without CB history, *SpA+CB+* patients with SpA and CB history. For numerical data, see Table 3. All four variables were significantly lower in group SpA−CB− compared to both SpA+ groups. Sclerosis score and sclerosis depth were significantly higher in SpA−CB+ compared to SpA−CB−. BME score, sclerosis score, and depth were comparable between SpA−CB+ and SpA+CB−, whereas global assessment score was significantly lower in SpA−CB+. Sclerosis and global assessment scores were significantly lower in SpA−CB+ compared to SpA+CB+. No significant difference is seen for all 4 variables between SpA+CB− and SpA+CB+. *BME* bone marrow edema

**Fig. 5:**
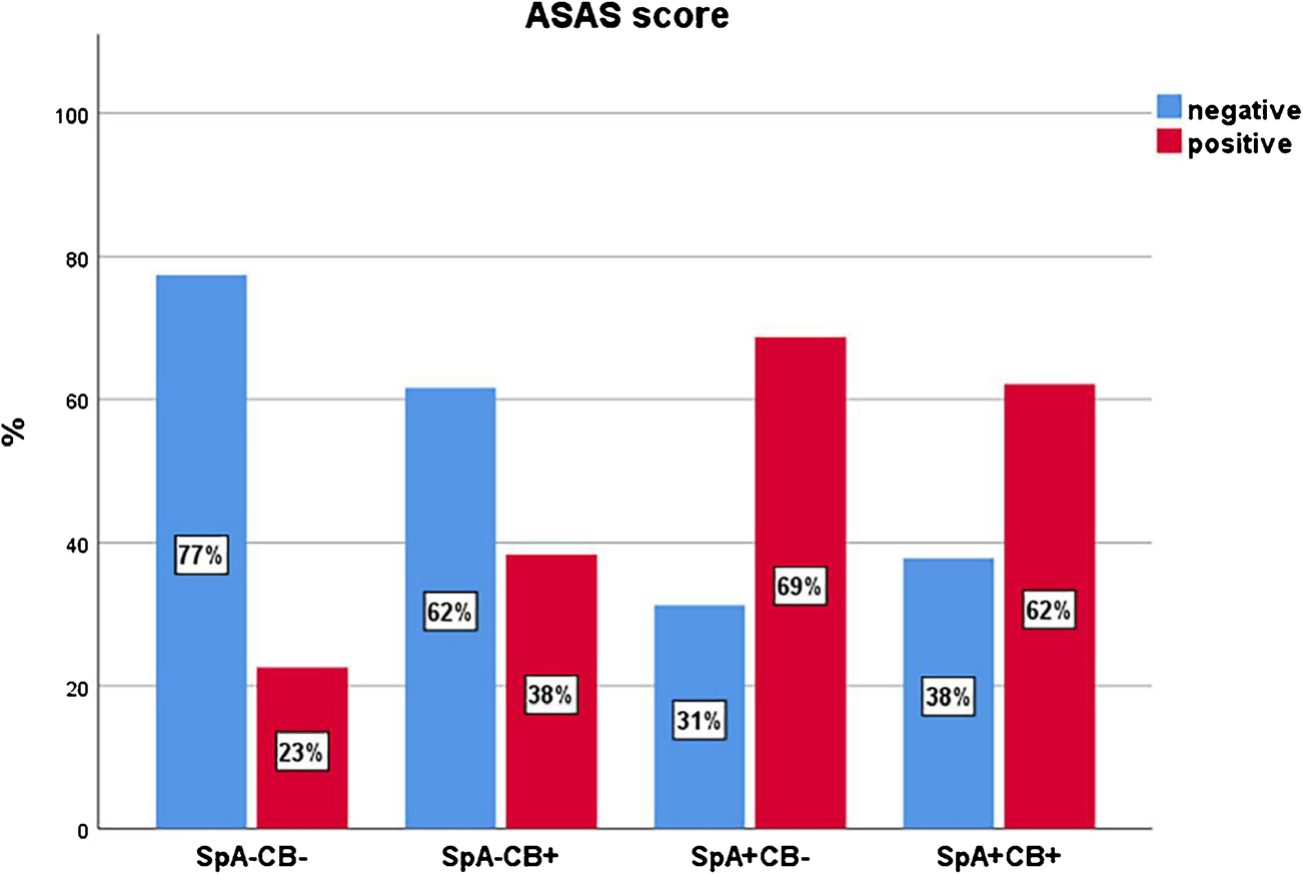
Bar chart illustrating ASAS score as either negative (blue) or positive (red) in the four different subgroups: *SpA*−*CB*− no SpA and no CB history, *SpA*−*CB+* no SpA with CB history, *SpA+CB*− SpA without CB history, *SpA+CB+* patients with SpA and CB history. ASAS positive for axSpA is defined as either one BME lesion on two or more consecutive slices or more than one BME lesion on a single slice [17]. No significant difference in ASAS score is seen between both SpA+ groups. All other 5 in between group comparisons are significantly different (for numerical data, see Table 3). *BME* bone marrow edema

Detailed numerical data for all 6 subgroup comparisons are shown in Table 3. BME score, sclerosis score, sclerosis depth, global assessment score, and ASAS score in each subgroup are illustrated in Figs. 4 and 5.

**Table 3:**
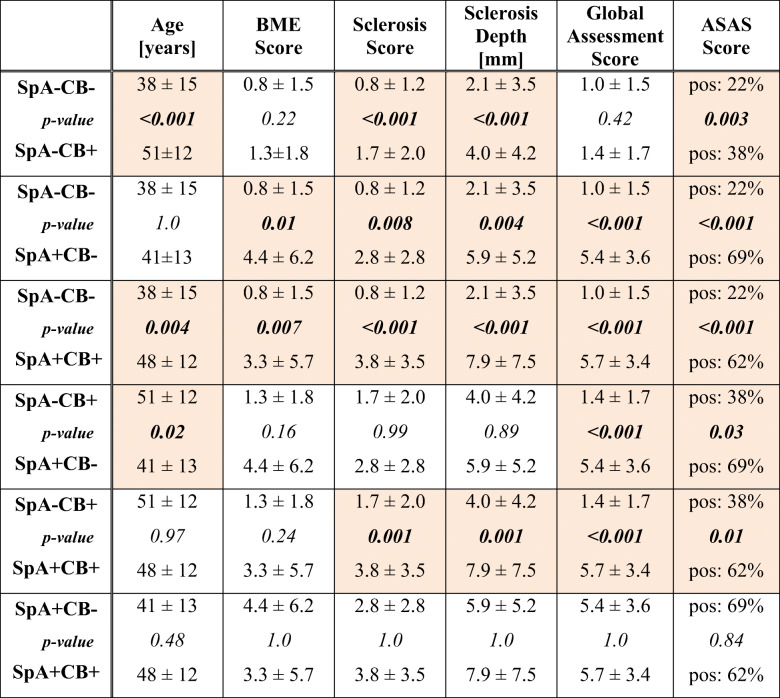
Subgroup comparison for age, BME, sclerosis, global assessment score, and ASAS score

Variables are given in columns; the six possible subgroup pairs are shown in rows. Numbers represent mean values ± standard deviation for continuous variables (age, BME score, sclerosis score, sclerosis depth, global assessment score). Percentages are given for the ASAS score. *p* values are shown between the variables for each comparison. The color-highlighted values are statistically significant differences (*p* < 0.05) for each variable in the respective subgroup comparison

*ASAS* Assessment of Spondyloarthritis International Society, *BME* bone marrow edema, *CB* childbirth, *SpA* spondyloarthritis, *SpA+CB+* patients with SpA and CB history, *SpA*−*CB+* no SpA with CB history, *SpA+CB*− SpA without CB history, *SpA*−*CB*− no SpA and no CB history

#### Age

Age was significantly lower in group SpA−CB− compared to both SpA−CB+ (*p* < 0.001) and SpA+CB+ (*p* = 0.004). Additionally, patients in group SpA−CB+ were significantly older compared to group SpA+CB− (*p* = 0.02). The other subgroup analysis yielded no significant differences.

#### BME

The subgroup SpA−CB− showed a significantly lower BME score compared to SpA+CB− (*p* = 0.01) and SpA+CB+ (*p* = 0.007). The remaining subgroup comparisons showed no significant differences (*p* ≥ 0.16).

#### Sclerosis

Both sclerosis score and sclerosis depth were significantly lower in SpA−CB− compared to the other three groups (*p* ≤ 0.008). The sclerosis score and depth were significantly lower in SpA−CB+ compared to SpA+CB+ (*p* = 0.001). The remaining group pairs showed no significant differences (*p* ≥ 0.89).

#### Global assessment score

Both SpA+ groups showed significantly higher scores compared to both SpA− groups (*p* < 0.001). No significant difference was seen between SpA+CB− and SpA+CB+ (*p* = 1.0) as well as between SpA−CB− and SpA−CB+ (*p* = 0.42).

#### ASAS score

Both SpA+ groups showed significantly more ASAS positive SIJ-MRI compared to both SpA− groups (*p* ≤ 0.03). Additionally, SpA−CB+ had significantly more ASAS positive SIJ-MRI compared to SpA−CB− (*p* = 0.003).

### Correlation analysis in subgroup SpA−CB+ (*n* = 193)

Table 4 illustrates the influence of age and childbirth-related factors on BME, sclerosis, overall assessment score, and ASAS score.

**Table 4:**
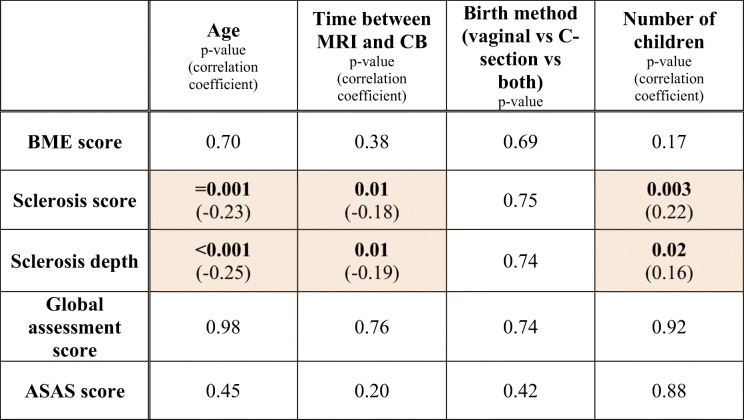
Correlation analysis in group SpA−CB+

Numbers represent *p* values. If *p* value is < 0.05, the correlation coefficient is given in parenthesis. The color-highlighted values are statistically significant correlations

*ASAS* Assessment of Spondyloarthritis International Society, *BME* bone marrow edema, *CB* childbirth, *C-section* cesarean section

#### BME

No association was found between age and BME score (*r* = 0.028, *p* = 0.70), time since last childbirth and BME score (*r* = 0.063; *p* = 0.38), birth method and BME score (eta = 0.11; *p* = 0.69), and number of children and BME score (*r* = 0.10, *p* = 0.17).

#### Sclerosis

A weak correlation was observed between age and total sclerosis score (beta = − 0.23, *p* = 0.001; regression coefficient = − 0.038; R-squared 0.054) as well as age and total sclerosis depth (beta = − 0.25, *p* < 0.001; regression coefficient = − 0.088; R-squared 0.062). In addition, there was a weak correlation between time since last childbirth and total sclerosis score (beta = − 0.18, *p* = 0.01; regression coefficient = − 0.002; R-squared 0.033) as well as between time since last childbirth and total sclerosis depth (beta = − 0.19, *p* = 0.01; regression coefficient = − 0.005; R-squared 0.035).

There was no significant association between birth method and total sclerosis score (eta = 0.055; *p* = 0.75) or total sclerosis depth (eta = 0.026; *p* = 0.74). However, the number of children showed a significant correlation with total sclerosis score (beta = 0.22, *p* = 0.003; regression coefficient = 0.45; R-squared 0.05) and with total sclerosis depth (beta = 0.16, *p* = 0.02; regression coefficient = 0.73; R-squared 0.027).

Tables 5 and 6 show results of multivariable regression analysis: Higher age was an independent predictor of both a lower sclerosis score (beta = − 0.43; *p* = 0.005) and a lower total sclerosis depth (beta = − 0.47; *p* = 0.002). Additionally, number of children was an independent predictor of a higher sclerosis score (beta = 0.25; *p* = 0.001) and a higher total sclerosis depth (beta = 0.20; *p* = 0.005). However, both models poorly predicted the effect on total sclerosis score with R-squared = 0.12 and total sclerosis depth with R-squared = 0.11 (independent variables: age, time since latest childbirth, number of children).

**Table 5:** Multivariable regression analysis in group SpA−CB+: influence on total sclerosis score

Influence on total sclerosis score				
Variable	Beta (unstandardized)	Beta (standardized)	Standard error	*p* value
Age (years)	− 0.07	− 0.43	0.025	0.005*
Time since last CB (months)	0.003	0.21	0.002	0.18
Number of children	0.51	0.25	0.14	0.001*
R-squared	0.12			

Multivariable regression analysis: effect of age, time since last CB, and number of children on total sclerosis score. Asterisk denotes significant *p* values (< 0.05)

*CB* childbirth, *SpA* spondyloarthritis

**Table 6:** Multivariable regression analysis in group SpA−CB+: influence on total sclerosis depth

Influence on total sclerosis depth				
Variable	Beta (unstandardized)	Beta (standardized)	Standard error	*p* value
Age (years)	− 0.17	− 0.47	0.054	0.002*
Time since last CB (months)	0.006	0.24	0.004	0.13
Number of children	0.87	0.20	0.31	0.005*
R-squared	0.11			

Multivariable regression analysis: effect of age and time since last CB on total sclerosis depth. Asterisk denotes significant *p* values (< 0.05)

*CB* childbirth, *SpA* spondyloarthritis

#### Global assessment score

No association was detected between age (*r* = 0.002, *p* = 0.98), time since last childbirth (*r* = 0.02; *p* = 0.76), birth method (eta = 0.08; *p* = 0.74), number of children (*r* = 0.008, *p* = 0.92), and global assessment score.

#### ASAS score

No correlation was found between age and ASAS score (*r* = 0.05, *p* = 0.45), time since last childbirth and ASAS score (*r* = 0.09, *p* = 0.20), and birth method and ASAS score (phi = 0.09, *p* = 0.42) or between number of children and ASAS score (*r* = 0.01, *p* = 0.88).

### Distribution of BME and sclerosis along the SIJ

**Fig. 6:**
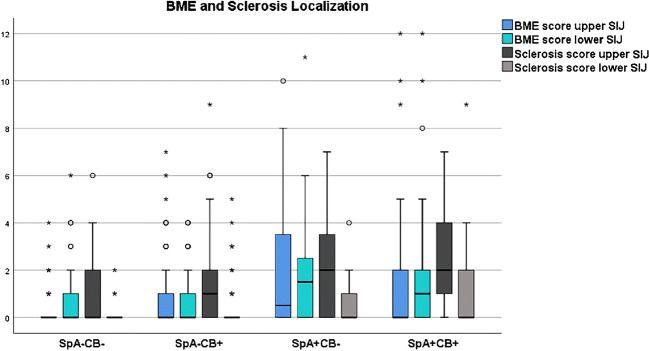
Boxplot illustrating localization of BME and sclerosis in the upper or lower half of the cartilaginous portion of the sacroiliac joints for subgroups *SpA*−*CB*− no SpA and no CB history, *SpA*−*CB+* no SpA with CB history, *SpA+CB*− SpA without CB history; *SpA+CB+* patients with SpA and CB history. In each subgroup, BME is distributed equally in the upper and lower portion of the sacroiliac joint, whereas sclerosis is predominant in the upper portion of the sacroiliac joint in each subgroup. *BME* bone marrow edema

BME score was equally distributed in the upper and lower half along the cartilaginous portion of the SIJ for all four subgroups (*p* > 0.22), whereas subchondral sclerosis revealed a marked predominance in the upper half of the SIJ in all four subgroups (*p* < 0.006) (Table 7 and Fig. 6).

**Table 7:**
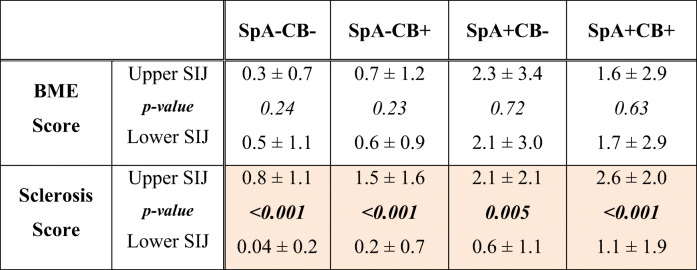
Comparison of BME score and sclerosis score between the upper and lower half of the sacroiliac joint within each group

BME is equally distributed in the upper and lower half of the sacroiliac joint for each group, whereas sclerosis score is significantly higher in the upper half of the sacroiliac joint compared to the lower half for each group. The color-highlighted values are statistically significant differences between the upper and lower SIJ

*CB* childbirth, *SpA* spondyloarthritis, *SpA+CB+* patients with SpA and CB history, *SpA*−*CB+* no SpA with CB history, *SpA+CB*− SpA without CB history, *SpA*−*CB*− no SpA and no CB history

## Discussion

The purpose of this cohort study was (1) to investigate the long-term (more than 24 months after latest childbirth) effect of pregnancy/childbirth on SIJ regarding BME and subchondral sclerosis on MRI in comparison to SpA-related changes and (2) to test if “birth method” and “number of children” are predictors for the extent of BME and sclerosis.

In concordance with the current literature, SpA patients—irrespective of childbirth—showed significantly higher BME scores compared to non-SpA patients without childbirth history [7, 10, 27]. However, our study comprised 193 non-SpA patients with an average timespan of 21 years between MRI and last childbirth (range 2-63 years), thereby providing new insight in the SIJ-MRI appearance of non-SpA patients in this broad late postpartum period. In our cohort, pregnancy/childbirth yielded no significant effect on BME score. This indicates that the well-known effect of pregnancy/childbirth on SIJ regarding BME in the early postpartum period [9–11, 14, 16] will vanish with time, which is consistent with findings of a recent study by Hoballah et al., comparing women without childbirth history with women in either the early (first postpartum year) or late postpartum period (≥ 2 years after delivery) [16]. We found no association between either age, time after last childbirth, birth method, or number of children and BME score in our cohort.

Subchondral sclerosis occurred more frequently and was more pronounced in non-SpA patients with childbirth history (64%; average total depth 4.0 mm) compared to non-SpA patients without childbirth history (40%; average total depth 2.1 mm), which is consistent with the findings in other publications [6, 14, 16]. Interestingly, our study showed no differences in regard to sclerosis score or sclerosis depth between non-SpA patients with childbirth and SpA patients without childbirth, whereas significant differences prevailed between non-SpA patients with childbirth and SpA patients with childbirth. These findings indicate that childbirth alone can cause subchondral sclerosis, not only in the early postpartum period [9, 11, 14, 16], but also persisting as chronic subchondral sclerosis in the later postpartum period. Hoballah and colleagues recently described the occurrence of SIJ sclerosis not only in the early but also in the late postpartum period (range 2-28 years after last childbirth) [16]; however, our cohort offers a broader postpartum range from 2 to 63 years after last childbirth and a direct comparison to SIJ-MRI findings in SpA patients with or without childbirth history.

We found both “age” and “number of children” to be independent predictors of subchondral sclerosis: Higher age was an independent predictor of a lower total sclerosis score and lower total sclerosis depth, whereas higher number of children was independently associated with a higher total sclerosis score. These findings imply that a longer duration of SIJ being exposed to mechanical stress during pregnancy and/or childbirth leads to more subchondral sclerosis on SIJ. Interestingly, the birth method (vaginal delivery or C-section) does not seem to influence the extent of subchondral sclerosis. Regarding sclerosis detection, we acknowledge the fact that CT is more sensitive compared to MRI [28]. However, detection of erosions and joint space changes (e.g., ankylosis) as typical structural changes seen in SpA can be diagnosed with similar accuracy in MRI compared to CT [28] and, most importantly, the ability of MRI to detect active sacroiliitis (osteitis/BME)—as opposed to CT—makes MRI more appropriate and practical for early diagnosis of SpA in daily practice.

Additional structural changes along SIJ (ankylosis, erosions, fat metaplasia) were not systematically analyzed in our study, but evidence suggests that ankylosis and erosions are very rare in non-SpA patients [11, 29] and fat metaplasia in general with its various appearances (focal, patchy, confluent) is a frequent finding in non-SpA patients, increasing with age [4, 16, 29]. However, we used a “global assessment score” including all possible active (osteitis/BME) and structural (ankylosis, erosions, fat metaplasia, backfill, sclerosis) SIJ changes related to SpA to express the overall diagnostic confidence level for SpA diagnosis based on MRI. As one might expect, a significantly higher diagnostic confidence level was seen in both SpA groups compared to both non-SpA groups. A positive childbirth history in general on the other hand seems not to cause sufficient long-term SIJ changes to lead the radiologist towards a higher suspicion of SpA. In other words, when all possible active and structural SIJ lesions on MRI (osteitis/BME, ankylosis, erosions, fat metaplasia, backfill, sclerosis) are taken into account, the fact “positive childbirth history” in general seems not to influence the radiologist’s interpretation/classification of a SIJ-MRI as SpA or non-SpA.

Interestingly, we found an equal distribution of BME in the upper and lower half of SIJ in both SpA patients and non-SpA patients, whereas sclerosis was found predominantly along the upper SIJ in both SpA patients and non-SpA patients, suggesting localization of sclerosis seems not to be a helpful diagnostic feature in distinguishing SpA-related sclerosis from pregnancy/childbirth-related sclerosis on SIJ.

Our study has limitations. First is the retrospective study design with its inherent drawbacks, e.g., no asymptomatic control group and the lack of follow-up, meaning a false-negative or false-positive classification regarding SpA cannot be ruled out with certainty for each included patient. Nevertheless, by excluding those patients with either inflammatory back pain but no fulfillment of the ASAS criteria for axSpA [17] or mechanical back pain but MRI findings highly suspicious for axSpA, we can confidently assume an accurate classification within our cohort. Second, osteoarthrosis as another cause for subchondral sclerosis and/or BME was not systematically taken into account in the analysis, potentially confounding our results; however, during MRI analysis, there were no signs of unequivocal osteophytes as a sign of clear osteoarthritic changes of SIJ. Moreover, scarce history was available regarding potential confounders such as other forms of mechanical stress on the SIJ, e.g., physical activity. Additionally, patients without SpA and with childbirth history were significantly older compared to patients without SpA and without CB history; however, age showed no significant correlation with either BME score or sclerosis score in the entire cohort (*n* = 349). Although two different magnetic field strengths have been used with slightly different slice thickness, within the subgroups, the portion of 1.5 T and 3 T MRI datasets was almost identical; hence, an impact of this variable on the results is highly unlikely. The strength of our study is the substantial sample size (*n* = 349) and detailed obstetric history with a large timespan between MRI and last childbirth, representing the actual heterogeneity in patient populations of daily clinical practice. Furthermore, SpA diagnosis of all included subjects has been confirmed by a board-certified rheumatologist.

In conclusion, our study indicates that pregnancy/childbirth may cause persistent long-term subchondral sclerosis on SIJ with a predominance along the superior portion of the cartilaginous SIJ, which can be indistinguishable from SpA-induced changes. In contrast, BME adjacent to SIJ—which is known to be present in the early postpartum period—seems to resolve over time and should not be considered a pregnancy/childbirth-related MRI finding in the later postpartum period. Time since last childbirth is not an independent predictor for subchondral sclerosis. Birth method yields no effect on sclerosis of SIJ; however, more pregnancies/childbirths seem to result in increased extent of sclerosis on SIJ.

## Abbreviations

ASAS: Assessment of Spondyloarthritis International Society
axSpA: Axial spondyloarthritis
BME: Bone marrow edema
CB: Childbirth
C-section: Cesarean surgery
ICC: Intraclass correlation coefficient
SIJ: Sacroiliac joint(s)
SpA: Spondyloarthritis
SpA−CB−: Subgroup without SpA and without childbirth history
SpA−CB+: Subgroup without SpA and with childbirth history
SpA+CB−: Subgroup with SpA and without childbirth history
SpA+CB+: Subgroup with SpA and with childbirth history
STIR: Short tau inversion recovery
